# Using Racial Justice Principles in Medical-Legal Partnership Design and Implementation

**DOI:** 10.1017/jme.2023.159

**Published:** 2023

**Authors:** Alice Setrini

**Affiliations:** 1: LOYOLA UNIVERSITY CHICAGO SCHOOL OF LAW, CHICAGO, IL, USA

**Keywords:** Racial Justice, Mlp Design, Implicit Bias, Systems Thinking

## Abstract

Medical-legal partnerships (MLPs) have the potential to address racial health disparities by improving the conditions that constitute the social determinants of health. In order to live up to this potential, these partnerships must intentionally incorporate seven core racial justice principles into their design and implementation. Otherwise, they are likely to replicate the systemic barriers that lead to racialized health disparities.

The reality of longstanding racial health disparities in the United States can no longer be ignored. The Black, Indigenous and other communities of color that are disproportionately impacted by poor social determinants of health continue to have worse health outcomes than their white counterparts.^2^ These multidimensional challenges require interdisciplinary solutions, and medical-legal partnerships (MLPs) are designed for just that purpose.

The need, however, to explicitly center communities of color and their lived experiences in implementing health-promoting solutions is often overlooked in the design and function of traditional MLPs, an oversight that can lead to recreating and perpetuating many of the same structural barriers to health equity that exist within healthcare infrastructure generally.^3^ Including the core principles of racial justice advocacy as designed by the Shriver Center on Poverty Law’s Racial Justice Institute (RJI) in the development and operation of MLPs not only improves the structure and function of these interdisciplinary partnerships, but also provides community-driven mechanisms for creating systemic change.

This paper first explores how understanding structural racialization, systems thinking, and implicit bias provides the vocabulary and foundational understanding for creating MLPs grounded in racial justice advocacy principles. It then describes how engagement in community lawyering, multi-form advocacy, intentional framing and communication, and internal organizational alignment, builds on that foundation for an MLP prepared to effectively address racial health disparities.

## Core Principles of Racial Justice Advocacy

Racial justice advocacy and training is by no means a new concept; civil rights and legal service organizations have been thinking in these terms for decades.^4^ Advocates, however, trained primarily in issue spotting and problem-solving, often seek to implement solutions without thoroughly understanding the history, culture, ideology, and interconnected nature of the institutions and policies that shape the communities they serve. In 2014, the Shriver Center on Poverty Law launched the RJI, a formalized training program and service provider network that centers racial justice in legal services.^5^ Integral to RJI are the seven core principles of racial justice advocacy: understanding structural racialization, implicit bias, and systems thinking; practicing community lawyering, multi-form advocacy, effective framing and communication; and creating internal organizational alignment.^6^ Grounding legal services practice in this framework provides advocates with tools for understanding how their clients experience systems and structures that disproportionately harm people of color, and supports advocacy aimed at dismantling those structures. This same framework can be used in designing MLPs to target legal solutions aimed at improving racial health disparities.

Systems thinking is the practice of understanding how systems work by identifying their component parts, analyzing how they intersect, and assessing the results they produce. It is a perspective that looks at relationships and interactions rather than seeking linear, reductive, or causal frameworks to explain outcomes. To produce outcomes that are racially equitable, MLP practitioners and program designers can use systems thinking in MLP design to determine where and how to intervene in the various intersecting systems that affect patient and community health.

### Understanding Structural Racialization

Most health care and legal practitioners are aware of structural and systemic barriers to health from decades of data collection and analysis, which consistently demonstrate the nature and extent of racial health disparities in the US.^7^ Structural racism, or structural racialization, is the way society fosters racial discrimination through mutually reinforcing systems like housing, education, and employment, that then leads to policies and practices that reinforce discriminatory beliefs, values and outcomes.^8^ To appreciate the influence structural racialization has on the healthcare system and the outcomes it produces, it is necessary to acknowledge not only how these racialized health disparities are produced, but also that underlying racialized sensibilities themselves, both conscious and unconscious, create our sociopolitical structures.^9^ These structures in turn affect how we see ourselves and the communities we serve. Racial disparities in healthcare persist in spite of decades of research and interventions in part because until recently, we have failed to understand that race affects the foundation and operation of our healthcare institutions. This reckoning is necessary to build legal tools for addressing health disparities that do not reinforce and reproduce the very structures they aim to dismantle, and is part of a movement that is occurring in both legal and medical education.^10^

MLP advocates seek to address health-harming social needs, which have a vastly disproportionate impact on communities of color, through systemic legal interventions.^11^ To do this, MLP providers must move past an understanding of racism as a psychological condition, attitude, or prejudice held by individuals. Much of the literature on the health consequences of discrimination, as well as the contemporary understanding of racism, focuses on interpersonal experiences.^12^ Recognizing and defining the interconnected layers of internalized, interpersonal, institutional, and structural racism allows MLP practitioners to identify, analyze, and address these issues in their programs, both with their health care partners and in their own advocacy.

While terminology can vary, *internalized racism* generally refers to that which occurs within oneself, and is expressed as internalized feelings of superiority or entitlement among the dominant constituent social group, or of inferiority among members of Black, Indigenous, and People of Color (BIPOC) communities; *interpersonal racism* refers to directly perceived discriminatory interactions between individuals; and *institutional racism* refers to discriminatory policies and practices that occur within institutions.^13^ *Structural racism* is the racial bias that exists between and among institutions and across society. It is the ecosystem where all these subordinate levels of racism interact to reinforce discriminatory beliefs, values, and distribution of resources.^14^ With this common vocabulary, we can conceptualize how the distinct layers of racism manifest in our health and legal systems, impacting individual and community health, and creating and exacerbating health disparities.

### Systems Thinking

Systems thinking is the practice of understanding how systems work by identifying their component parts, analyzing how they intersect, and assessing the results they produce. It is a perspective that looks at relationships and interactions rather than seeking linear, reductive, or causal frameworks to explain outcomes.^15^ To produce outcomes that are racially equitable, MLP practitioners and program designers can use systems thinking in MLP design to determine where and how to intervene in the various intersecting systems that affect patient and community health. The principles of systems thinking can guide how MLP practitioners take contemporary racial conditions into consideration when responding to obstacles that inevitably occur when addressing health disparities.

Every system consists of interrelated parts that work together to produce particular outcomes.^16^ There are three primary components to a system: the parts and players involved in the system; the relationships and interactions between the various parts; and the outcomes the system produces (whether intentional or not). MLPs, like systems found in nature (the human body), as well as human-made (city transit systems) include these components. MLPs can differ in their parts and players, for example, depending on the type of health care partner involved, specific patient population addressed, or health issue in focus. Because a system is more than just the sum of its parts, however, its behavior often cannot be described or predicted merely by looking at its individual components. The more complex the system, the more the relationship between its parts may influence its behavior. These relationships can also impact the outcomes produced by a system, which may differ from what its players would identify as its purpose.

For example, in an established MLP, the providers might say the purpose of a referral system requiring patients to proceed through a centralized intake unit is to efficiently direct patient-clients to intake staff. In practice, however, this system may create a gatekeeper effect that stops potential clients from accessing legal advocates. Using a different referral mechanism, such as one that provides a “warm handoff” from medical provider directly to an attorney, might take more effort initially (e.g., because it requires gathering permissions to share patient information and building the infrastructure to accept referrals directly), but adding this component may actually produce a preferred outcome for the system. MLP designers can use systems thinking to identify the purpose of their MLP system, or the outcomes that they want to produce, and then work backwards to build and implement the structures needed to achieve those outcomes. This is not the typical framework for legal advocacy, which usually reacts to broken systems by developing strategies and tactics to intervene.

Legal advocates and healthcare practitioners often have limited understanding of problems based on where they are situated within a system. For example, a medical provider may understand that a patient that tests positive for lead poisoning needs to either remediate their apartment or move. However, that provider may not know whether that family receives a housing subsidy for that apartment, if that subsidy allows for transfer to a new unit, what the payment standard is for that subsidy, whether there are any units available at that payment standard level in the school system where the older children in the household are enrolled, and so on. This is not to say the medical provider needs to have this level of understanding for every challenge a patient may have, but it exemplifies how systems thinking requires examination of the system as a whole and from many different perspectives. This reveals how each part of the system may be influencing others, and when MLP practitioners engage in this systems analysis as a team, both in relation to systems outside the MLP, as well as within the MLP, they improve each player’s capacity to analyze problems and improve outcomes.

Finally, the most important part of a systems thinking analysis when the aim is addressing racial health disparities is to see how different racial and ethnic groups fare within the system. This is vital when assessing MLP function. Who is most burdened by the system? Who is most advantaged? Answering these questions enables an MLP to collect good data and feedback on whether a chosen intervention is aligned with the community the program serves, designed to minimize any harmful effects, and advances the racial justice goals identified by the stakeholders.

Using systems thinking in MLP design moves away from the typical approach of observing a symptom and treating it independently, which leads to short-term and often unintended outcomes. A systems approach requires the sometimes-tedious work of uncovering the root causes of surface-level issues. Only by understanding the short and long-term relationships between the parts of a system can we design solutions that maximize racially equitable outcomes.

### Social Cognition and Implicit Bias

Implicit bias reinforces and supports the phenomenon of structural racialization. This is reflected in the social assumptions and structures that result in racially disparate health outcomes. Although most healthcare providers hold no *explicit* biases and express a desire to treat patients of all races equally, they still engage in differential treatment of patients based on race.^17^ Implicit bias at various stages of a medical encounter, as well as during a legal intervention on the part of providers and patient-clients, affects how both parties hear, see, and respond to each other.^18^ This can lead to poor adherence to recommendations and follow-through, as well as provider mistrust, which, in turn, contributes to racially disparate health outcomes. It is crucial for MLP practitioners to have a strong grasp of how implicit bias can influence provider to patient-client interactions, and to employ bias mitigation techniques to address health disparities through their programs.

Neurotypical brains organize and categorize information into schemas, which are memory structures that organize new information and create mental shortcuts to make sense of the enormous amounts of information a human being encounters every day.^19^ Social knowledge, which is also organized and stored in these schemas, is imported from culture and environment through music, television, social media, family, friends, and so forth. Social cognition — the generalization of social characteristics — develops from these sources and can be either positive or negative. Associations strengthen over time, becoming automatic, and implicit bias is born. Scientists say that within five hundred milliseconds of identifying a person’s race or ethnicity, we associate that person with the most dominant association stored in our brains.^20^ Regardless of whether this is a positive or negative association, the process is still harmful; this unconscious association devalues the individual by reflecting assumptions irrespective of their accuracy and ultimately directs how we interact with the outside world.

Two potential manifestations of implicit biases are stereotype threat and racial anxiety; both have an impact on healthcare.^21^ Stereotype threat refers to the pressure people feel when they fear their performance may confirm a negative stereotype about their group. It is triggered in situations where an individual who is not part of the dominant culture or in tune with its norms wants to blend in with the majority and avoid association with negative stereotypes. Legal service organizations, health care organizations, and MLPs all develop cultural norms and value systems, which, when not intentionally cultivated, can develop to reflect dominant culture mental models, creating stereotype threat for individuals who fall outside the dominant culture. The impacts of stereotype threat are reduced cognitive capacity, lowered expectations, and dis-identification from the stereotyped group, as well as anger and depression.^22^ Racial identity anxiety is the discomfort experienced or anticipated when engaging with people from other racial or otherwise distinguishable groups.^23^ Non-dominant group members fear they will *experience* bias in the form of discrimination, hostile treatment, or invalidation of their experiences, while dominant group members fear they will *exhibit* bias against those in non-dominant groups.^24^ This leads to general unease and the desire to avoid interracial contact. In the context of a medical or legal service encounter, it also contributes to a biased care model, where bias permeates both patient-client and provider conduct and communications, negatively affecting patient satisfaction, compliance with instructions, adherence to advice and treatment, and trust.^25^

Fortunately, effective mechanisms for reducing the impact of bias in our encounters exist. Patterns and habits acquired over a lifetime and reinforced through culture are difficult to break, so techniques must aim to consciously override these behaviors. Increasing bias awareness, exposure to counter-stereotype individuals, and the creation of environments that empower community members who are not part of the dominant culture have all been shown to reduce bias. In particular, techniques shown to mitigate bias include purposeful adoption of the perspective of a wide range of stakeholders, (e.g., development of fair and clear criteria for decision making processes); making time for deliberative processing that formalizes decision making and seeks input from others; reducing cognitive load; stressing the importance of individuation, the conscious consideration of each individual’s identity beyond their racial or otherwise distinguishing group; and developing effective mechanisms for accountability.

Designing and running an MLP presents many scenarios where bias can intrude. However, breaking down major design decisions into smaller choice points and utilizing targeted interventions where biases are triggered can mitigate the impact of bias in the program. Examples of these tactics include conducting trainings for all stakeholders, using Implicit Association Tests^26^ to create awareness, and establishing an environment that empowers community members both visually and practically by incorporating programs and activities that connect service providers with persons of other races, ethnicities, and class status as peers, rather than only in contexts with inherent power imbalances. This is particularly challenging in the context of an MLP, where both medical and legal service providers are already stretched thin with responsibilities, and power imbalances are inherent in services provided. Reorienting our priorities to focus on racial justice requires creating opportunities to engage with patient-clients on equal footing. This combination of self-reflection and incorporation of bias mitigation techniques will go a long way towards reducing the impact that implicit bias has on services, which can, in turn, reduce health disparities.

These three components of racial justice advocacy — understanding structural racism, systems thinking, and implicit bias — provide a theoretical groundwork for practical tools to use when designing and implementing an MLP. Mechanisms for advocacy that center the lived experiences of MLP clients and community-based solutions designed to improve health equity are also critical.

## Practice Elements of Racial Justice Advocacy

### Community Lawyering

Traditional legal services typically provide a menu of legal issue areas where advocates have expertise; if the available services meet a client’s needs, the client might receive representation, depending on institutional capacity.^27^ This results in a system where client issues must be triaged so only those in a crisis situation that meets the priorities of the legal service provider have access to representation. MLPs focus on addressing issues upstream, in order to alleviate this challenge.^28^ A community lawyering model — where the lawyer has an expansive view of their role, a deep understanding of the community served and its leadership, and a goal for improving the environment for that community that originates from within that community — takes this concept one step further, allowing MLP advocates to look to patient-client communities for the interventions and supports needed to promote health. When combined with human-centered design thinking, an MLP can disrupt the traditional mechanisms for legal service provision and center the goals of patient communities collectively facing racial health disparities. Many MLPs focus on a specific population or a particular legal or health issue.^29^ To avoid creating an MLP that provides a solution in search of a problem, the affected community should play a lead role in the decision-making process to determine an MLP’s scope of service and focus. An example of community lawyering can be seen in the Health Forward/Salud Adelante MLP, where the population focus for the MLP were patients who received care coordination services from Cook County Health in neighborhoods where a strong network of community partners already existed.^30^ Focus groups and community-led discussions highlighted the health-promoting legal services that were most needed were for formerly incarcerated citizens and unhoused individuals. As a result, a subsidiary project was created to address public benefits needs specifically for those individuals who were unhoused, had substance use disorder, or were referred from the social work team at the county jail deferment program.

Partnering with patient-clients to design their own health-promoting solutions, where the advocate’s role is essentially that of an advisor and technical assistant, takes time, effort, relationship-building, and trust. This model of shared leadership empowers communities and creates stakeholder buy-in to the solutions the MLP will support. Creating space for feedback and evaluation, as well as building in funding for compensated focus group research to determine which tools and resources patients need to maximize health, are techniques for incorporating community lawyering into the MLP. Embedded patient leadership and feedback mechanisms, such as the patient board member model required of Federally Qualified Health Centers, as well as formal evaluation and accountability checkpoints, are pieces of community lawyering that all MLPs should consciously build into their structures.

### Multi-Form Advocacy

Direct client services, community legal education, provider training, collaborative resource creation, and multi-sector policy initiatives with broad coalitions are important multi-form advocacy tools for MLP implementation. However, it is critical to understand that not every MLP can or should be designed to “do it all”: mission creep is a very real challenge in environments with limited resources. That said, designing an MLP with multi-form advocacy in mind allows the program to identify and engage with additional coalition partners and address legal challenges as they arise. A classic example of multi-form advocacy within MLP is where a provider and/or advocate recognizes a trend or pattern of referrals and engages with coalition partners to address the problem via a policy intervention. This was the case with providers at Erie Family Health and the Health Justice Project at Loyola University Chicago.^31^ After seeing patients with lead poisoning who lived in subsidized housing and were unable to move into new, safe units without risking their subsidies, the partnership built a multi-sector, broad-based coalition to push the Department of Housing and Urban Development to update its federal lead regulations, with the patient community leading the initiative.^32^

### Framing, Communication, and Organizational Alignment

Because MLPs are interdisciplinary by nature, they bring together a wide variety of stakeholders with diverse backgrounds and expertise. Best practices require MLP designers and practitioners to use a form of cultural translation to engage in effective communication regarding the improvement of health outcomes for the communities served. Legal advocates are generally well-versed in taking technical procedural legal language and simplifying it for non-legal audiences.^33^ Medical providers also do this in the clinical or hospital context every day. Combining data on health outcomes with humanizing storytelling creates a powerful tool for MLP practitioners to engage in the types of multi-form advocacy described.^34^ By allowing patient-clients to tell their own stories in their own words, and to drive advocacy initiatives through a community asset-based framework that centers resilience and power, MLPs can serve as narrative-shifting mechanisms for communities of color. With so many stakeholders involved in creating a successful MLP, organizational alignment is vital to maximize effectiveness in addressing health disparities. This alignment requires clear communication of specific goals, with sufficient participation from individual stakeholders to maintain the mission’s momentum. Selecting health and community partners and ensuring that a critical mass of internal MLP stakeholders stand behind the goals and strategies identified through systems thinking processes and community lawyering techniques are ways that organizational alignment manifests in MLP design.

Most of these concepts are not novel to current MLP practitioners and designers; they likely engage in many if not all of these core principles at some level already. However, a purposeful focus on race when applying these advocacy tools to MLP design and operation is necessary to move the needle on racial health disparities. We need tools to help us better understand the complex systems that create health disparities, along with community driven solutions that meaningfully advance racial equity and justice rather than simply replicating familiar patterns and practices and hoping that this time, things will be different. When focusing on community through this racial advocacy framework, MLPs can be an effective part of the solution.

## Note

The author has no conflicts to disclose.
